# Impulsive Insanity.—The French Vampire
*Communicated by G. Sigmond, M.D., F.S.R.S., formerly Professor of Materia Medica to the Royal Medico-Botanical Society, and Lecturer on Medical Jurisprudence, Grosvenor School, St. George's Hospital.


**Published:** 1849-10-01

**Authors:** 


### Original Communications.

### IMPULSIVE INSANITY.—THE FRENCH VAMPIRE.*

A MOST striking and appalling instance of insanity has just occupied the attention of the French psychologists. It has called forth the talents of Brierre de Boismont, of Michea, and of several other celebrated mental pathologists. It is one of those cases which, whilst it shocks the feelings of all those who have the ordinary susceptibilities of human nature, is accompanied with such disgusting traits of unnatural sensation, that many of the points must still be left in mystery, as they are scarcely fit even for medical discussion. It is true that our neighbours are less delicate on such subjects than we are, and that the scientific journals that have narrated the occurrences have entered with a minuteness upon them that would scarcely be palatable to our own more sensitive tastes; and they have ransacked both ancient and modern records to furnish forth similar proofs of moral depravity.

Towards the close of the year 1848, a rumour was prevalent in Paris that awoke the most fearful alarm. It was whispered about that the cemeteries of the dead had been broken into; that bodies lately interred had been dragged from their gloomy receptacles and savagely mutilated. To a people so singularly devoted to respect the remains of their friends, who take frequent opportunities of visiting their graves, and who, more particularly amongst the humbler classes, are accustomed on certain days to hang upon the tombs

* Communicated by G. Sigmond, M.D., F.S.R.S., formerly Professor of Materia Medica to the Royal Medico-Botanical Society, and Lecturer on Medical Jurisprudence, Grosvenor School, St. George's Hospital.

memorials of their affections, and to strew around, as was formerly customary in England, flowers and herbs, this information was most harassing. Every care was paid to the due watching of the cemeteries, and, if possible, to ascertain what motives could lead to the perpetration of such acts. It was evident that it was not that love of science which formerly led medical men in search of anatomical knowledge, for the bodies that had been exhumed had been treated with violence. Limbs had been torn off, the internal parts had been disembowelled, yet with one or two exceptions there had been no portion missing.

On the 16th of November in that year, Dr. Pujol was summoned by the commissioner of police, of the quarter of the Luxembourg, to attend at the cemetery of Mount Parnassus, and to draw up a medical report upon a case which demanded medico-legal investigation. He found there the body of a female, aged about fifty; the corpse was lying upon its back at a little distance from the tomb, surrounded by large cypresses; it was partly covered by its winding-sheet, so that the head and the lower extremities only were visible; the right angle of the mouth had been cut asunder by a sharp instrument almost to the ear; a deep incision had been made in the throat. On taking off the covering, two very large mutilations came into view; the whole of the interior part of the body was laid open by an immense incision, which embraced the whole length of the thoracic and abdominal cavities. The limbs exhibited still greater mutilations; the right arm had been dragged from its socket and placed between the thighs, which had also undergone disarticulation; the various tissues had been hacked by some instrument; nothing was to be seen which could in any way account for what had occurred. There had been no attempt to take away the clothing—the examination had not been scientific—there had evidently been much labour employed in reaching the body—there was no one who, from motives of revenge, could have been led to the violation of the sanctity of the dead; in short, the whole was involved in a mystery which no one could unravel.

The subject had scarcely ceased to form the daily conversation of the neighbouring coffee-houses, when a similar occurrence gave rise to the greatest agitation. On the 12th of December, the same physician was sent for to the same cemetery for a precisely similar object to that which had so lately demanded his attention. Upon this occasion, it was the corpse of a female, still young, that he was called upon to inspect, and to draw up a report. The body, which had been taken from its coffin, had been dragged about fifty paces from the grave. One incision was to be noticed, which it was evident had been performed for scientific purposes, and upon inquiry at a later period, the hospital in which she had died attested the fact; but besides this, there were none of those savage mutilations which before were visible. Here, for the first time, was publicly noticed that the gratification of some horrid propensity must have urged the perpetrator of these deeds upon his frightful expedition. The publicity given by the journals to these shocking events brought to light several cases which had been whispered about, but which had not become the object of inquiry. It was made known by the Mayor of Ivry, that at that village, which is close to Paris, there had been occurrences of a similar nature, which had baffled every inquiry, and which had thrown the surrounding neighbourhood into the utmost consternation.

A young girl, of the name of Gillet, had been buried amidst the lamentations of her friends, who had been shocked a few days afterwards by the discovery that her corpse had been dragged from its resting-place; the stomach and the bowels had been opened. A female, a married woman, Charpitelle, who died in childbirth, had also been similarly treated thirteen days after her interment. The body had been opened, the heart had been taken away, but all the other organs were left in their normal state. In these instances there was an erotic manifestation. The putrifying remains exhibited that there had been an attempt to perpetrate crime, and to gratify an unnatural appetite. Disgust and horror followed upon these crimes. In the public mind there existed a general feeling of dismay at the idea of being compelled to bury a relation in places where such atrocious deeds were committed. The mystery which enveloped the whole matter added to the alarm. The guardians of the cemeteries were doubled and trebled; watch-dogs and blood-hounds were employed; spring-guns, infernal-machines, steel-traps, were set, and the utmost publicity given to the steps that were taken for securing the catacombs and cemeteries of the dead. All seemed in vain. The guns were heard apparently discharging of their own accord; the infernal machines were found to have gone off; but although there were even observed traces of blood, and marks that deep injuries had been inflicted upon the intruder, there was no solution to the mystery—there was no one discovered. The cemetery of Mount Parnassus had evidently been scaled on the 15th of March, 1849. The machine, which had been charged with all sorts of missiles, had gone off; the invader had evidently not escaped unhurt; his blood remained as evidence that he had been wounded; but the watchmen who rushed to the spot had found no one, and they were in a state of despair, when one of those accidental circumstances which occasionally seem to be destined to reveal obscure crimes, took place, and enabled the hand of justice to seize the perpetrator of these unseemly and disgusting acts.

Two soldiers of the seventy-fourth of the line were stationed as sentries at the cemetery of Mount Parnassus, on the occasion of the execution of the men who killed General Brea, during the insurrection in June. They entered into conversation with the guardians of the place, and in talking of the severity of wounds, they mentioned those of one of their comrades, Serjeant Bertrand, who had gone into the military hospital on the night of the 15th of March, having been, according to his statement, attacked by some of the insurgents whilst returning to his barracks. They dwelt upon the number of the wounds, and the singular way in which they had been inflicted, as well as the nature of the missiles that had been employed. The narrative awoke the curiosity of the listeners, who, comparing the time when the attack was said to have been made, and the peculiarities attendant upon the injuries that had been inflicted, arrived at the conclusion that they had gained a clue to the long wished-for discovery. They communicated their suspicions to the medical attendant of the wounded man, M. Marchal, of Calvi, who, acting with great prudence and discretion, waited until Serjeant Bertrand was out of danger, and then gradually unfolding to him the nature of the accusations that were brought against him, and for which he was to be tried before a court-martial, obtained from him a complete revelation of his disgusting deeds, and a confession of all the circumstances connected with them, establishing in the mind of his humane and scientific physician a conviction that a monomania of a frightful character had possession of him, and that he was urged on to the commission of horrors and of crimes by an impulsive insanity, which left him entirely without control over his passions and his actions.

By the 10th of July, Serjeant Bertrand was sufficiently recovered to be able to appear before the council of war of the department of the Seine for the purpose of undergoing his trial for the misdemeanor of which he was accused. It was a matter of much regret that he was to be placed before a military tribunal, not only as far as the satisfaction of the public mind was concerned, but also in reference to the information which was expected to accrue from the investigation to medical and psychological science. But as Paris was at the period under military law, the whole of the department of the Seine being in a state of siege, the ordinary courts of law could have no cognizance of delinquencies committed by soldiers. The investigation, however, was well conducted, and the presiding officer was most anxious that the truth should be elicited. If, however, medical opinions were not there sufficiently examined, the publications of Brierre de Boismont and others have left nothing to desire; and there has been as much attention devoted to the subject as is necessary for the complete investigation of the state of mind of the individual. When Serjeant Bertrand made his appearance in the court, every eye was naturally directed towards him, and the preconceived opinion from what had been brought forward in the charge, led the spectator to expect to see an unprepossessing person, with a countenance of ferocious and unpleasant expression. The surprise was therefore the greater, on seeing a young and interesting man, leaning on crutches, and evidently suffering under the pain of recently-inflicted wounds, advance with an air of respectful deference and gentle manner. He appeared to be about twenty-five years of age; his figure of the middle height, thin, and well-proportioned; a marked and well-defined profile of Grecian character; a fine forehead; his eyes full of life and expression, with something of a melancholy cast; his hair chesnut colour. The phrenologist could discover nothing in the form of the head that could indicate sensuality or brutality; on the contrary, without studying minutely the organization, he would pronounce him to be intellectual, whilst the physiognomist would find sufficient of energy, yet of mildness and benevolence, in the expression of his countenance, to say that he could do nothing contrary to the laws of society or to the Divine revelation—at least, that he would not be guilty of those ferocious acts which lead to the idea of innate wickedness, and which exclude their perpetrator from society. On being questioned by the president, he unhesitatingly acknowledged the commission of the deeds of which he was accused, and for which he was arraigned before the tribunal. He had drawn up a statement, he said, of what had occurred, and which he requested his physician to read before the court. He did not seem in the slightest degree affected by the situation in which he stood; listened with apparently a deep interest to the document; now and then looking round the court, as if he expected to find sympathy from his audience, and as if he considered himself rather as a martyr or a sufferer than a person accused of crimes which are visited by public execration.

His confession began by stating, that, as early as seven years old, there had been a species of madness observed in him, but that it had never led him into any excess. He had satisfied himself by walking in the most gloomy recesses of a forest, where he had remained for several days plunged in the deepest gloom; but it was not until the 23rd of February, 1847, that a kind of fury seized him, and led him on to the execution of those acts for which he was then in a state of confinement. He then described his first paroxysm. He was walking in the country with one of his comrades,—for he was then in the army, having studied originally for the church, but suddenly enlisted, and rapidly promoted to the rank of serjeant,—they passed a cemetery, which curiosity induced them to enter. A person had been buried the previous evening; the grave-digger, surprised by a storm, had not filled up the grave, but had left his tools upon the ground. At this sight, fearful ideas crossed his mind, a violent headache and a beating of the heart came on; he seemed to lose possession of his faculties; he made a pretext to return into the town. As soon as he had got rid of his companion, he returned to the cemetery, laid hold of the shovel, and dug up the grave. Scarcely had he taken the body out of the earth, than he began to beat it with the shovel in a rage, such as he has never been able to account for; but when he saw a peasant approaching the gate of the cemetery, he laid himself down by the side of the corpse, and remained there some moments: the peasant, who had also seen him, ran to acquaint the authorities of it; he got out of the grave, restored the body, covered it with earth, and escaped over the wall. He was then trembling all over, a cold sweat covered his body; he hid himself in a small copse in the neighbourhood; and notwithstanding a heavy rain which continued to fall, he laid down and remained from twelve to three in a complete state of insensibility: on his recovery from this condition, his head was in a feeble state, and his powers of body diminished. The same thing occurred after each act of madness. Two days afterwards, he returned to the cemetery at midnight during a heavy storm of rain. Finding none of the implements, he tore up the same grave with his hands; they were soon covered with blood, but he was insensible to pain. He dragged out the body, he tore it piecemeal, and threw back the portions into the grave, which he filled up with his hands. Four months then elapsed; he fancied that he had become calm, he thought that his madness was terminated; when upon going to Paris, some of his friends invited him to accompany them to the cemetery of Père la Chaise. The sombre walks delighted him; he determined to go there during the night, and at nine in the evening he scaled the walls, walked there, agitated by sombre visions, for half an hour. He then began to disinter a body without any implements. He delighted in the joy of tearing a body in pieces. He then returned home. This was in the month of June. For fourteen or fifteen days he followed the same course, when he was surprised by two of the watchmen of the cemetery, who were preparing to fire upon him; but as he had always carefully filled up the graves after the mutilation, they had observed nothing, and he got out of the scrape by saying that having been intoxicated, he had entered the cemetery; that he had laid down under a tree, and fallen asleep until that moment. They obliged him to leave the place, but nothing else occurred, though the risk that he had run made such an impression upon his mind that for seven or eight days afterwards, he avoided returning to the cemetery. The events of February occurring, his regiment quitted Paris, and did not return until the affair of June, when the disease developed itself with redoubled violence. He was stationed at the camp at Ivry; and during the night, although the sentinels were numerous and vigilant, and the discipline severe, he contrived to pass out; nothing could stop him. Every night he managed to reach the cemetery of Mount Parnassus, where he acknowledged that he delivered himself up to every species of excess. The first victim of his paroxysm was a young female, whose limbs he mutilated and threw about. This profanation took place about the 25th of July; since that he returned only twice to the cemetery. The first time it was a lovely moonlight night; he saw a watchman armed with a pistol patrolling. He was hid in a tree near the outward wall, which he had scaled for the purpose of entering the cemetery; the man passed close to him, but he escaped unobserved, and he then got away without making any attempt to enter. The second time, he disinterred an old woman and a child; he treated these corpses precisely as he had done others. He then went to the cemetery where suicides, and those who die in the hospitals, are buried, continuing the same treatment of dead bodies. The first person he there exhumed was a man who had been drowned, and whose body he opened; this was towards the end of the month of July. One of the most remarkable facts, to which, of course, much interest is attached, and which he himself observes that he never was able in any way to account for, was, that he never attempted to commit any sort of mutilation upon the corpses of the men whom he disinterred; but, on the contrary, that he cut the bodies of females in pieces with sensations of exquisite pleasure. On one occasion, the 6th of November, 1848, he disinterred two male and two female bodies, mutilating the females only. On this occasion, as he was scaling the walls of the cemetery, a pistol was fired at him, but he escaped uninjured. This did not discourage him; he laid himself down, notwithstanding the inclemency of the weather at the time, and slept at least two hours. He then penetrated into the burying-ground, disinterred the corpse of a young girl that had been found drowned, and mutilated it. From this period up to the 15th of March, he only twice revisited the scene of action; once on the 15th of December; the other occasion, the beginning of January. On both these intrusions, he found the guardians on the alert, for each time he was fired upon. The ball on the first occasion nearly touched him, having traversed his great coat behind; the second did not come near him. He examined the position of the machine from which missiles were thrown, and remarked the manner in which it was discharged, and took precautions to escape its effects. From this period up to the 15th of June there was no return of the paroxysm; on the contrary, he felt an estrangement from that peculiar sensation, which he describes as having for some time formed his enjoyment. At the time that he made the attack which led to his discovery, he was free from his excitement; but the feeling of curiosity induced him, as he was passing the cemetery of Mount Parnassus, to scale the wall, and just at the moment that he reached the parapet, and was about to jump down, he received the injuries, for the cure of which he was admitted within the walls of the hospital. He declared, that had he on that occasion not been so severely wounded, he should never again have attempted to enter a burial-ground, for that his boldness had completely left him. During the first period of his excesses, he never committed them unless at times when he had taken somewhat freely of wine; but latterly he required no such stimulus to call his morbid appetite into play; any disagreeable circumstance occurring to him urged him on to the committal of these acts. He added that it might be imagined, after all that had been stated, that he might also have a desire to injure the living; on the contrary, he was exceedingly gentle towards all mankind, and he could not do mischief to a child. He felt persuaded that he had not a single enemy, and that all the non-commissioned officers were attached to him for his frankness and for his gaiety.

Thus ended the confessions made by this singular being, upon whom every eye was fixed with earnest scrutiny, which he stood with modest demeanour and unabashed look. He appeared to have little or no compunctious visitings, and to be perfectly at his ease during the whole time that the long document was read by his medical attendant. A pause of some moments followed, and then there was almost a breathless silence, when M. Marchal, of Calvi, stated that he had revelations of a most important character to detail; that he made them with the perfect consent of the serjeant. It had been previously believed that the insanity of the unfortunate young man was connected with cannibalism; that he was urged on to his atrocities by a love of devouring human flesh; and that he had fed upon the hearts and upon the bowels of some of the corpses. As this species of unnatural propensity has been known to exist, there was an idea that allusion was to be made to such a form of mental aberration; but the horror of the audience, of the judge, and of the court was fearfully awakened as the physician unfolded the sequel. He began by observing that those who had heard what had been read would no doubt have been struck by the singular allusion that had been made to the preference shown by the accused to female bodies; the profanations had been accompanied by still further atrocities. The exhumation of the bodies had been for an end; the mutilations had been only accessories—cohabitation with the dead had been the object. When the corpse of a man was disinterred by him, he felt a loathing and a disgust; but when a female presented itself, he rushed upon it with avidity and ardour. He satisfied a deplorable passion, and then fell into a state of convulsive stupor. He laid down upon the ground in the open air, amongst the shrubs, let the weather be of the most severe kind. He remained in a lethargy unconscious of all that passed around him, and for several hours seemed lost to every earthly feeling. It would be useless to enter into minute details, or to follow the learned physician further. There was sufficient evidence of the facts that were advanced; the foul depravity of the man's taste was acknowledged by himself, and the annals of the medical profession furnish similar histories; fortunately they are of exceeding rarity. The sexual desire seems to have been instinctively produced by the sight of the corpse; whether it was the irresistible impulse produced by this depraved passion that led to the exhumation, in the first instance, seems somewhat doubtful, though this opinion is generally held by the psychologists, who consider that the increased energy of the frame that rendered him capable of surmounting every difficulty, and braving every danger, was the result of the inordinate development of the erotic passion, which accidentally was perverted to a depraved taste. The repulsive story seemed borne out by all the facts that were produced, and the man's declaration was looked upon as conclusive evidence, therefore the court did not pursue the investigation any further; it felt incompetent to decide upon the nature of the mental delusion. It contented itself, therefore, with declaring the man guilty of a misdemeanor; for which the highest punishment inflicted by the Code Napoleon is twelve months' imprisonment. However unsatisfactory this amount of punishment may appear, either for the chastisement of such hideous deeds, or for the protection of society against their repetition, no other course could have been followed; but upon the expiration of this term, the police will exercise for some time a surveillance over him, and should any circumstance lead to the belief that the monomania still exists, he will be consigned to confinement for life in the Bicêtre, or some lunatic establishment, as the evidence of his physician will now be a sufficient ground for taking ulterior measures.

The discussion excited by the opinions of the medical men are interesting in the highest degree; but that which is entertained by Michea is the one generally prevalent, that the maniac was urged on by a depraved amatory feeling; that which M. Marchal, of Calvi, has promulgated, that the organ of destructiveness was developed beyond its normal state, whilst the erotic passion was only an accessory, has not gained much ground. Those who have argued upon the probability of the correctness of the first proposition, have adduced numerous extraordinary instances of the perversion of the moral faculties, in consequence of a depraved generative instinct. They refer to the evidences that exist of this degradation of human nature in the annals of antiquity, and point to the secret museum in the palace at Naples, and to the discovery that was made at Pompeii, of a room devoted to scenes which surpass in their iniquity anything that modern invention has dared to execute; and Brierre de Boismont has even gone further in his illustrations; he has brought forward instances, one occurring in his own practice, in which the sexual passion, which is certainly in some instances one of the most imperious governors of the whole organization, has been directed towards the commission of immoral, cruel, and indecent acts. In the middle ages, the chroniclers have given to us narratives which exhibit the wild ravings of madmen, upon whom satyriasis, erotomania, and nymphomania had exerted their baleful power, and led them to the commission of atrocious folly, which the legal tribunals of the day associated with sorcery and witchcraft, and condemned the miserable wretches to the torture and to flames. The descriptions of Delancre, of what occurred by those who believed themselves possessed by the agents of Satan, and driven by him to the consummation of every frightful act of depravity, have been the theme of much examination by those whose duty it is to study every form of insanity, and who are compelled, with a view of assisting in the due administration of the laws of their country, to examine subjects which neither correspond with their taste nor with their morals; and show that the perversion of the sexual instinct exists, and as frequently leads to impulsive insanity, as do suicidal, homicidal, or incendiary monomania. Doctor Castelnau has, in "La Gazette des Hopitaux," shown that the annals of medical literature furnish us with instances which prove that the disease of Serjeant Bertrand has been developed in other individuals; and although there is no case where there was a long continued series of crimes committed, there can be no doubt that there are well authenticated facts which substantiate the matter. It will be remembered that Georget, in his "Examen Medical des Procès Criminels," gives us the case of Leger, an old soldier, who was tried in the year 1824, by the Court of Assizes at Versailles, who one day seized a little girl at the entrance of the forest, murdered her, sucked her blood, violated her, tore out her heart, and mutilated her bosom and her sexual organs, besides eating some portion of her flesh. Upon his trial, he wore an air of gaiety and satisfaction, until he heard the melancholy evidence of the poor girl's mother, when he burst into tears, and with much apparent sympathy, exclaimed, "I am, indeed, sorry to have deprived you of your daughter. I implore your pardon." Georget, who examined the case minutely after the man had been condemned to death, (which sentence he heard with the most perfect indifference,) says of him, "He was not, as has generally been said, a great criminal, a monster, a cannibal, a man eater, who wished to renew the feast of Atreus; this individual was, in my opinion, an unfortunate imbecile, a madman, who ought to have been confined in the Bicêtre as a lunatic." That Bertrand was a cannibal certainly is not proved; but that a depravity of taste, requiring for its satisfaction human flesh collected from the foulest sources, sometimes occurs, there can be no doubt whatever; and Dr. Bartholett has given us an instance of a man of thirty, who sought from the most disgusting and filthy carcases his nourishment, disinterring corpses that had been long buried, and feasting upon them.

Amongst those who have written upon the case, there are several who have confounded depravity of manners with depraved instincts, and have cited cases which show that in all ages there have been individuals who have forgotten that their passions were given them to fulfil the noblest duties of existence, and who have indulged in sensuality until they have vitiated their tastes, and disunited the gratification of the mind from the body, which totally annihilates all the higher feelings of our nature, and places us on a level with the brute creation. One of the writers in the "Gazette Medicale de Paris" has most injudiciously attempted to connect the hideous propensity of Serjeant Bertrand with the state of the military life, and has taken the pains to bring together numerous instances of the immoral conduct of several soldiers, whose cases have from time to time drawn attention. That celibacy, the frequent separation from the other sex, may be the cause of inexcusable delinquencies, it is not necessary to deny; but it would be most absurd to attribute them to aberrations of intellect, and the ingenious arguments of the clever author who has undertaken the loathsome task of recording vice and folly, will not rank him amongst psychologists and mental pathologists, who are anxious to ascertain what may be considered punishable offences, and what may be safely pronounced instances of insanity.

Physicians have a task of the utmost difficulty to fulfil, they are to discover whether there are not special and decided symptoms, not perceptible to ordinary observers, which form the true indication of insanity; they have been accused by high authority of introducing a new word into criminal jurisprudence, that of monomania; as Regnault declared, for some years "at every assize court an excuse has been set up for crime," the advocate employs it in every desperate cause, the physician regards it as a new path by which he can obtain reputation, and juries only hear it as another source of incertitude, and another reason for embarrassment in their sacred functions. It is, therefore, incumbent on all those who can throw light upon the subject, to render the doctrines of the day more complete, more certain; to assist in that classification upon which the whole basis of the study of mental disease rests. Besides which, there is an important point to be ascertained—Can any mental force—we will call it education—be so directed to bear upon the intellectual organization, that it shall be enabled to withstand those irresistible impulses which lead to the commission of unexpected and unusual acts? Can we see the incubation of such disease? or shall we not by study be hereafter enabled to do so? Shall we not, by watching the progress and development of the human mind, be capable of imparting to it such force and healthy vigour as will make it withstand the predominance of any one ruling passion, and by giving the power of self-control, whether by religious or moral instruction, teach energy to the slumbering reason, and so invigorate it as to rescue it from impending disease? It is singular that in this case, as well as others of a somewhat similar character, the instinctive desire to commit an act contrary to the laws of God, of nature, and of men, should not be associated with any conception of its enormity—not the slightest appearance of self-accusation or of repentance for what had been perpetrated, seemed to cross the mind of the guilty person; he had scarcely any feeling of shame; he was surprised at the reproaches others heaped upon him; and when an attempt is made to awaken some other sentiment within him, it produces no effect; all considerations are swallowed up by the momentary whirlwind of the passion; he is carried to his object; neither the tempest of rain, the bitter cold, the painful exertion, nor the fear of being killed, influence him; he is insensible to everything during the paroxysm; and even during the intermission he has no proportionate horror at his propensity. All these facts demand earnest investigation. Are they in themselves arguments irresistible? Is insanity the only explanation of them? There seems to be no premeditation, no concealment, except when instantaneous punishment is apprehended,—no fears for personal safety, no dread of punishment,—all yield to the engrossing impulse which is daringly executed, and frequently repeated. Would not education or watching over him when he gloomily walked in the woods have prevented the exhibition of his mental aberration? Have not the greater part of the histories of monomania shown some moment when the careful psychological observer would mark the coming on of disease? These are points which must yet be deeply and carefully studied by the mental psychologist, for as yet we have no clue to their explanation. I must here introduce a case, which made in France, some years since, an indelible impression upon mental pathologists; it is but little known in England, but ought to be recorded in our literature. The circumstances were narrated at the time only in the "Gazette des Tribunaux." On the 2nd of July, in 1828, about ten in the evening, three young girls in a village in France were sitting by the side of the river, bathing their feet, when Nicholas Rousselot, a man living in the village, came up to them and tried to lay hold of them. The two most active escaped; the third, Genevieve Barroyer, had not time to do so. Forced to walk into the stream, she called out to the man, "Do not be foolish; we are in the water." He, without answering, took her and threw her down. The river was not very deep. The companions of the young girl ran, frightened, into the village, and spread the alarm; a great number of persons ran quickly to the spot, but it was too late, the body of the girl was found by the side of the water spread out, all her garments were thrown over her head, and twisted in such a way as to have prevented her respiration and the use of her arms; a large contusion was visible upon her chest, evidently produced by the pressure of the knee. Rousselot had disappeared as soon as the persons came rushing down the river, and remained concealed two days and two nights in a neighbouring wood, and on the third day he delivered himself up from hunger. He was tried at the assizes for the murder; the greater number of the witnesses testified that he was a man of whom all the females in the neighbourhood were in constant dread; he seemed always tormented by sexual desire; not only had he made several attempts upon the young women of the village, but all sorts of strange stories were afloat about his conduct to the women throughout the whole department; he was supposed to be somewhat deranged since he had been ill the previous year. The man appeared about thirty years of age, small in size, a thin visage, with deep sunk eyes and pallid look. During the trial, he kept his eyes constantly fixed on the ground, without evincing any emotion. Those who knew him from his infancy, described him as of a sombre, savage character, always avoiding society, and of obstinate taciturnity. The medical men who had charge of him declared that he never evinced any mark of insanity. Besides the murder of this girl, he was accused of an attempt on a married woman shortly before. The accusation was, that he had murdered the girl, and then attempted to violate her; the defence was, insanity; upon both points he was found guilty, but by one of those decisions which lead the public to believe that the jury is more insane than the man they are trying, they considered that he had committed the murder in a state of insanity, but that he was in his senses when he committed the act of violation. This inexplicable enigma of judicial logic still remains as a psychological problem. The accused was condemned to be shut up for ten years for the minor offence, and afterwards to be committed to a lunatic asylum. Verdicts of this kind show the necessity for more minute knowledge, and for more accurate judgment, in the judicial investigation of psychological crimes.

---

### MADNESS, AS TREATED BY SHAKSPERE.

